# Sequential thrombosis and bleeding in a woman with a prolonged activated partial thromboplastin time

**DOI:** 10.1186/1477-9560-9-16

**Published:** 2011-10-27

**Authors:** Akpan Spencer, Michael I Pearce, Paul RJ Ames

**Affiliations:** 1 Department of Medicine, Airedale Foundation Trust, Steeton, BD20 6TD, UK; 2 Department of Haematology, Airedale Foundation Trust, Steeton, BD20 6TD, UK

**Keywords:** thrombosis, acquired haemophilia, pregnancy, lupus anticoagulant

## Abstract

Simultaneous or sequential haemorrhage and thrombosis in the presence of a prolonged activated partial thromboplastin time (aPTT) is a rare occurrence: we describe the case a 37 year old lady who developed post-delivery deep vein thrombosis treated with low molecular heparin and warfarin followed a week later by extensive bruising over legs and forearms, a significant drop in haemoglobin and a very prolonged aPTT. Further tests revealed an acquired factor VIII inhibitor at 35 Bethesda Units. We discuss the clinical and laboratory implications and provide a literature review of simultaneous thrombophilia and haemophilia in the presence of a prolonged aPTT.

## Background

The differential diagnosis of a prolonged aPTT (activated partial thromboplastin time) is manifold and necessitates an algorithm to indicate factor deficiencies or the presence of inhibitors that could be lupus like, heparin like or specific to a clotting factor [1]. A quick and correct laboratory diagnosis is of the utmost importance in the emergency setting when a clinician is faced with a critical haemorrhage in a previously healthy individual. We describe the sequential occurrence of thrombosis and haemorrhage developing over a nine day period in a young woman with a prolonged aPTT ratio.

## Case presentation

### Description

A 37 year old woman presented to casualty for pain and swelling in the right calf; her Well's score was 5, D-dimer elevated at 422 ng/ml (cut-off limit 230 ng/ml) and a Doppler ultrasound revealed a clot in the upper portion of the right femoral vein and in the deep veins of the right calf. With regards to thrombosis risk factors, she was 5 weeks post caesarean delivery, had been on oral contraception for the previous three weeks, had a body mass index of 24.5 (normal 18.5-24.9) had no varicose veins, and had not been on any long haul trips by either plane or car since delivery. Her personal history was uneventful, this being her first pregnancy during which she had no complications. There was no family history of thrombosis or recurrent miscarriages. Her aPTTr was 3.01 (normal range 0.88 -1.16) and prothrombin time ratio (PTr) was normal. Her Hb was 9.1 g/dl with a slight microcytosis. Low molecular weight heparin (LMWH) at treatment dose was started embricated with warfarin then stopped after four days when her international normalised ratio (INR) was 2.3. A week later she re-attended casualty for spontaneous bruising on upper limbs and worsening of the pain and swelling in the right leg that was markedly ecchymotic. Blood tests on admission revealed Hb 8.1 g/dl, platelets 557 × 10^9^/L and C-reactive protein at 247 mg/L (normal < 5 mg/L). Urine and blood cultures were negative. Since 1988 in Airedale the possibility of a bleeding disorder was tested by comparing the clotting times of two aPTT reagents, currently Synthesil (IL) as the reagent sensitive to factor inhibitors and deficiencies and Actin FS (Dade) as the reagent insensitive to factor inhibitors and deficiencies but sensitive to factor XII deficiency [2]. Unbeknown to the laboratory personnel the same comparison can be used to detect a lupus anticoagulant [3]. Both assays are run on an automated coagulometer (TOP-CTS, Instrumentation Laboratories). The patient's INR was 2.3 (due to warfarin), the Synthesil aPTTr was disproportionately prolonged at 5.03 whereas the Actin FS aPTTr was 3.21 and a thrombin time was 12.8" (normal 12-16.5") ruling out a possible residual effect of LMWH that had been stopped three days earlier. Given the prolongation of both aPTTs, the laboratory personnel informed the consultant haematologist of a possible factor inhibitor and pursued factor assays that revealed a factor VIII at 2 IU/dl (normal range 60-180 IU/dl), factor XII at 65 IU/dl (normal range 50-180 IU/dl), factor XI at 61 IU/dl (normal range 60-140 IU/dl) and factor IX at 46 IU/dl (normal range 50-150 IU/dl). A factor VIII inhibitor was detected at 35 Bethesda Units. A repeat Doppler US of the right lower limb did not show thrombus in the femoral vein but extensive haematoma in the calf obscuring the possibility of persistent thrombus. The following day her Hb had dropped to 6.9 g/dl: warfarin was stopped and the patient was given a total dose of 8 mg of recombinant VIIa intravenously, repeated two hourly 12 times after transfer to the Haemophilia Centre at Bradford Royal Infirmary. The patient was started on oral cyclophosphamide 100 mg daily continued for six months and prednisolone 1 mg/kg for 6 weeks, gradually tapered down over the ensuing five months: three weeks into immune suppression her factor VIII levels was 36 IU/dl and in June 2010 her factor VIII was 62 IU/dl with normal aPTTs. A thrombophilia screen done at the same time (plasma levels of protein C, protein S, antithrombin and gene testing for factor V Leiden and prothrombin mutation, anticardiolipin antibodies and lupus anticoagulant) was negative. At 16 months follow up she is inhibitor negative with normal plasma factor VIII level.

### Discussion

Our patient shifted from deep vein thrombosis (DVT) to bleeding within the span of nine days: those involved in her care had not noticed that her aPTTr had been getting higher throughout her pregnancy (Table 1) and that it was markedly abnormal at her attendance for DVT. A factor VIII level at 2% and a factor VIII inhibitor at 35 Bethesda units were unequivocal with respect to pregnancy related acquired haemophilia: the latter is a life-threatening antibody-mediated haemorrhagic disorder that occurs prevalently in people over 50 years of age presenting with bleeding and/or with a prolonged aPTT. The FVIII inhibitor may develop during and/or after pregnancy with a prevalence of 7% to 21% according to series. It appeared in the first pregnancy in 80% (16 of 20) of women and did not seem to recur with further pregnancies [4-10] though in one survey it recurred in the three women who had successive pregnancies [11]. In general the inhibitor is identified on occasions of overt bleeding: in one series 50% of bleeding occurred in the peri-partum period and in 50% within 30 days of delivery; in another series bleeding occurred pre-partum in two women, within three days of delivery in three and within 3-12 months in nine [11,12]. Another review showed that 3% of 27 women bled during pregnancy, 3% after an abortion, 15% immediately after delivery, 30% within 4 months and another 30% between 4 to 12 months of delivery; this review also shows that post-partum factor VIII inhibitor often disappears spontaneously [12].

**Table 1:** Evolution of coagulation tests since late pregnancy

	2009		2010				Range
	Sept 30	Dec 31	Jan 2	Mar 3	Mar 10	Mar 12	
Gestational age (weeks)	**26**	**39**	**39**				
Puerperium (weeks)				**5**	**6**	**6**	
PTr	1.00	0.90	1.03	1.26	2.05	2.32	0.9-1.12
aPTTr	1.01	1.23	1.32	3.01	9.31	5.03	0.88-1.16
FNG (mg/dl)	638	> 700	816	538	701	691	150-400

Prothrombin time ratio (PTr), activated partial thromboplastin time ratio (aPTTr) and fibrinogen (FNG) from the end of the second trimester of pregnancy (September 30) to end of puerperium (Mar 12)

However, prior to haemorrhage, our patient had suffered a DVT, quite a rare occurrence in the presence of a bleeding diathesis. In the absence of genetic or acquired thrombophilia, four haemophilia A patients developed spontaneous limb vein occlusions [13-16], one with a duplicated superficial femoral vein [16] whereas a haemophilia A youngster who was also factor V Leiden heterozygous suffered a spontaneous cerebral infarction [17].

Conversely two haemophilia A patients with inherited thombophilia developed a fatal pulmonary embolism and a DVT after a surgical challenge [18,19] whereas a severe haemophilia A patient also factor V Leiden heterozygous suffered portal vein thrombosis after continuous infusion of F VIII [20]. DVT has been described also in a haemophilic A patient with factor VIII inhibitor 18 days after recombinant activated factor VII infusion [21].

With regards to acquired haemophilia, a small series described a spontaneous DVT in a 60 year old woman and in an 80 year old man with splenic marginal zone lymphoma and a proximal leg vein occlusion after a femoral to popliteal by-pass in a 76 year old man [22]; pulmonary embolism developed in a 29 year old man who was on tranexamic acid to minimise bleeding episodes that were managed with recombinant factor VII when occurring acutely [23]: none of these patients had inherited or acquired thrombophilia.

Within the thrombosis setting a prolonged aPTTr may indicate the presence of a lupus anticoagulant (LA). In the flurry of the Friday afternoon when the acquired factor VIII inhibitor was detected, whilst the patient was administrated the first dose of recombinant factor VII and then transferred to Bradford Royal Infirmary, we overlooked that the Actin FS aPTTr was shorter than the Synthesil aPTTr (3.21 vs 5.03): a study comparing sensitive and insensitive reagents to the LA showed that the shortening of the aPTTr with Actin FS (the insensitive reagent) can be suggestive of LA even in patients on oral anticoagulants and/or with acquired haemophilia [3]. Hence we could not rule out that our patient also had a LA that was not detected on subsequent testing because of the immunosuppressive treatment she received. Of the four reported cases where acquired factor VIII inhibitor coexisted with LA the clinical phenotype was always bleeding [24-27]. With regards to haemophiliacs with anti-factor VIII inhibitors, one study showed a simultaneous LA detected by the dilute Russell viper venom time (DRVVT) (and Staclot LA) in 12 such patients [28] whereas a positive LA was more common in haemophiliacs with anti-factor VIIII inhibitors than in haemophiliacs without inhibitors either by DRVVT (IL-LAC screen and confirm) (22% vs 10%) or by Staclot LA (Stago, France) (30% vs 5%).

## Conclusion

Thrombosis and bleeding are the yin-yang of haemostasis and their simultaneous occurrence must alert us to the rarity and complexity of such occurrences and induce us to have a sharp insight in these cases: acquired haemophilia A developing post-partum is not uncommon, a vascular occlusion on the background of acquired haemophilia A followed by bleeding aggravated by warfarin is unusual indeed. Given the differences in the Synthesil and Actin FS aPTT ratios, we cannot rule out that DVT was also promoted by a LA that might have disappeared after immune suppressive treatment: strict adherence to existing guidelines for LA testing [29] should have lead us to perform also a DRVVT (screen and confirm) to shed further light on the thrombotic event.

### Consent

The patient gave written informed consent for publication of this case report. A copy of the written consent is available for review by the Editor-in-Chief of this journal

## Competing interests

The authors declare that they have no competing interests.

## Authors' contributions

PRJA conceived the report and coordinated the manuscript that was drafted by AS and completed by MIP. All authors read and approved the final manuscript.
